# Cross-Sectional Analysis Investigating the Concordance of Maturity Status Classifications in Elite Caucasian Youth Tennis Players

**DOI:** 10.1186/s40798-019-0198-8

**Published:** 2019-07-01

**Authors:** Gillian K. Myburgh, Sean P. Cumming, Robert M. Malina

**Affiliations:** 1 National Tennis Centre, Lawn Tennis Association, 100 Priory Lane, Roehampton, London, SW15 5JQ UK; 2 Sport, Health and Exercises Science Research Group, Department of Health, University of Bath, Bath, UK; 3 Department of Kinesiology and Health Education, University of Texas at Austin, Austin, TX, USA

**Keywords:** Puberty, Adolescence, Youth athletes, Skeletal age

## Abstract

**Background:**

To evaluate the concordance of skeletal age (SA) with two predicted estimates of biological maturity status in elite British youth tennis players.

**Method:**

Participants were 71 male and female elite youth tennis players aged 8 to 16 years. Weight, height, and sitting height were measured. SA (Fels method) was the criterion indicator of maturity status. Maturity status was predicted with two methods: predicted age at peak height velocity and percentage of predicted adult height at the time of observation. Players were classified as late, average (on time), or early maturing with each method. Concordance of classifications was evaluated with kappa coefficients and Spearman’s rank order correlations.

**Results:**

Kappa coefficients between maturity status classifications were low in both sexes, − 0.11 to 0.22, while Spearman’s rank order correlations between maturity status classifications based on SA and the percentage of predicted mature height were moderate in males (0.35) and females (0.25), but the corresponding correlations based on predicted age at peak height velocity (PHV) varied, moderate and negative in boys (− 0.37) and low and positive in girls (0.11). Concordance of maturity status classifications based on the prediction methods and SA among tennis players was thus limited.

**Conclusions:**

Maturity status based on the percentage of predicted mature height at the time of observation correlated better with maturity status based on SA in contrast to status based on predicted age at PHV in this sample of elite youth tennis players.

**Electronic supplementary material:**

The online version of this article (10.1186/s40798-019-0198-8) contains supplementary material, which is available to authorized users.

## Key Points

- Associations between maturity status based on SA and percentage of predicted adult height at the time of observation were limited, but slightly better in males than in females.
- Associations between maturity status based on SA and predicted age at peak height velocity were poor.
- While indicators of maturity status based on somatic indicators are inexpensive, non-invasive, and practical, limitations of the methods should be recognized.
- It is prudent to consider additional indicators (e.g., more restricted chronological age groups, estimated growth rate, menarcheal status) when grouping youth tennis players for training and/or competition.

## Background

Maturity status (early, on time, late, mature based on skeletal age [SA], stage of puberty) refers to the state of biological maturation of an individual at the time of observation, while maturity timing refers to the ages when specific maturational events are attained (ages at peak height velocity [PHV] and menarche); though related, status and timing are not equivalent [1]. Individual differences in biological maturity status and timing impact body size, physical fitness, and athletic aptitude in youth [2] and are central to talent identification and development programs in many sports [3–5]. Available research on elite British youth tennis players, though limited, highlights a predisposition towards early maturing individuals [6, 7], which suggests that individual differences in growth and maturity status contribute to the identification and selection in the sport. Obtaining objective, reliable, and valid measures of biological growth (size attained) and maturity status would help inform identification and development strategies and perhaps increase the likelihood of long-term success [8, 9].

Established indicators of maturity status are SA and stage of pubertal development [4, 9, 10]. SA requires a standard radiograph of the hand-wrist, is a reliable and objective indicator of maturity status, and can be used from childhood through adolescence. There are three established methods for the assessment of SA—Greulich-Pyle, Tanner-Whitehouse, and Fels; though related, SAs with the three methods are not equivalent [4]. Secondary sex characteristics (stages of breast, pubic hair and genital development, testicular volume, pre- or post-menarche) are also reliable and objective indicators of maturity status but are limited to the interval of puberty [4]. Both SA and pubertal stages are often considered “intrusive” in that the former requires a small dose of radiation, while the latter are increasingly viewed as an invasion of personal privacy. Both methods also require trained and experienced assessors.

As a result, there is increased interest in the physical activity and sport sciences in two prediction methods, one of maturity timing and the other of maturity status: predicted maturity offset or the time before or after PHV, and percentage of predicted adult height attained at the time of observation [9, 11]. The former uses sex-specific equations which include age, height, weight, sitting height, and estimated leg length to predict maturity offset [11]. Chronological age (CA) at prediction minus offset provides an estimate of predicted age at PHV. Predicted maturity offset has been widely used to classify youth as pre-, circum-, and post-PHV (i.e., maturity status at the time of observation), but the protocol has been applied to samples of youth spanning relatively broad CA ranges and at times, to combined samples of boys and girls [12]. The maturity offset protocol is also increasingly applied in the context of talent development models and in the design and implementation of training programs [13].

Age, height, and weight of the youngster and midparent height are used to predict young adult or mature height [14]. The height of the youngster is then expressed as a percentage of predicted adult height, which is used as an indicator of maturity status [12, 15]. Among youngsters of the same CA, those closer to adult height are advanced in maturity status compared to those further from adult height. The percentage of predicted adult height at the time of observation is increasingly used in studies of youth athletes and physical activity [9, 12, 16–18].

Among male participants 9–14 years in American football [5] and 11–14 years in soccer [19], classifications of maturity status based on percentage of predicted adult height had moderate concordance with classifications based on SA; kappa coefficients varied between 0.11 and 0.50 and with the CA group. Among the soccer players [19], concordance of classifications of maturity status based on predicted ages at PHV and SA was also moderate, but kappa coefficients were low at 0.11 and 0.13. The study of soccer players did not classify players as pre- or post-PHV; rather, players were classified as late, average, or early based on the observed mean and standard deviation for age at PHV in the samples used to develop the prediction equations [19].

The concordance of maturity status classifications for participants in individual sports and for female youth athletes has not been systematically addressed. In this context, the purpose of this study was to evaluate the concordance of maturity status classifications based on SA, percentage of predicted adult height attained at the time of observation, and predicted age at PHV in cross-sectional samples of elite youth male and female tennis players. The central question of interest is whether currently available predictive methods for categorizing maturity status are appropriate among elite junior tennis players.

## Method

The sample included 71 elite British junior tennis players. Chronological ages, calculated as the difference between the date of birth and date of the skeletal age radiograph (see below), ranged from 9.88 to 15.95 years in 40 males and from 8.97 to 16.26 years in 31 females. The competitive status of the players and details of the study have been previously described [7, 20]. Based on self-ascribed ethnicity, the 71 players identified as Caucasian. Thirteen players (4 males, 9 females) included in the initial studies [7, 20] were non-Caucasian and were not included in the analysis as the prediction equations for two non-invasive maturity indicators were developed on Caucasian samples [14, 21]. The proportions of leg length and sitting height show ethnic variation [2], and the interaction of leg length and sitting height is a component of the maturity offset prediction equations [11].

Weight, height, and sitting height were measured on the first day of a 3-day National Age Group Camp held at the National Training Centre in Roehampton, London. The players were barefoot and wore shorts and a t-shirt; all headgear was removed. Weight was measured to the nearest 0.1 kg using a calibrated Marsden Weighing Company DP2400 BMI Indicator scale. Height was measured to the nearest 0.1 cm using a Harpenden stadiometer, with subjects standing with feet together, arms hanging relaxed at the sides, and head in the Frankfort horizontal plane. Sitting height was measured with the stadiometer; subjects sat on a 41-cm bench with their trunk upright, back against the stadiometer, and head in the Frankfort horizontal plane. Sitting height was the difference, to the nearest 0.1 cm, between height while sitting and height of the bench. Leg length was estimated as the difference between standing height minus sitting height.

A radiograph of the left hand-wrist of each of the players was taken onsite by a certified technician. Each radiograph was assessed with the Fels method for estimating SA [22] by an independent technician and an experienced assessor with the method. Details of the procedures have been reported previously [7]. The Fels method provides an estimate of SA and, in contrast to other methods of assessment, provides an associated standard error. Maturity status of each player was defined by the difference of SA minus CA. An SA within ± 1.0 year of CA indicated average or on-time maturity status, and a SA less than CA by < 1.0 year defined late status while a SA in advance of CA by > 1.0 year defined early status. The band of ± 1.0 year approximated standard deviations of SA with the Fels and other methods of SA assessment within single-year CA groups [23]. No player was classified as skeletally mature.

Maturity offset, the time before or after PHV, was predicted for each player with sex-specific equations developed on Canadian and Belgian youth [11]. The equations included interaction terms for estimated leg length and sitting height, CA and estimated leg length and CA and sitting height, and the weight by height ratio. CA minus maturity offset provided a predicted age at PHV which was used to classify each player as late, average, or early maturing relative to the estimated mean ages at PHV for the boys (13.8 years) and girls (12.0 years) upon whom the prediction equations were developed [2, 11]. A band of ± 1.0 year of the respective means was used to define average or on-time maturity status, 12.8 to 14.8 years in boys and 11.0 to 13.0 years in girls. The band of ± 1.0 year to define average maturity status approximated standard deviations for estimated ages at PHV in longitudinal studies [2]. Accordingly, a predicted age at PHV later than 14.8 years and earlier than 12.8 years classified boys as late and early maturing, respectively, while a predicted age at PHV later than 13.0 years and less than 11.0 years classified girls as late and early, respectively.

Young adult height was predicted with sex-specific equations developed on participants in the Fels Longitudinal Study [14]. The protocol required CA, height, and weight of the player and midparent height, the average of maternal and paternal heights [24]. The biological parents of each player reported their respective heights which were subsequently adjusted for overestimation [25]. The height of each player was then expressed as a percentage of his/her predicted adult height to provide an estimate of maturity status at the time of observation [15]. The percentage of predicted adult height for each player was converted to a *z*-score based on age- and sex-specific reference values for the longitudinal Berkeley Guidance Study of the University of California [26], as in other studies using the protocol with youth athletes [5, 9, 16, 17, 19, 27]. A *z*-score between − 1.0 and + 1.0 classified the player as average or on time in maturity status, and a *z*-score < − 1.0 classified the player as late, while a *z*-score > + 1.0 classified the player as early maturing [19].

Descriptive statistics (means and standard deviations) and cross-tabulations of maturity status classifications based on SA and the two prediction methods were calculated. As in the study of youth soccer players [19], concordance was assessed using percentage agreement and Cohen’s kappa [19, 28]; Spearman’s rank order correlations were also calculated. All analyses were performed using SPSS Statistics version 22.0 [29]. In addition, scatter plots of SA-CA differences and the *z*-scores for the percentage of predicted adult height and of SA-CA differences and the differences of predicted age at PHV and the sex-specific reference means were prepared. Of note, for both SA-CA and *z*-scores for the percentage of predicted adult height, differences of greater than − 1.0 and + 1.0 indicated late and early maturity status, respectively. In contrast, a negative score for the difference of predicted age at PHV minus the reference indicated early maturity status, and a positive score indicated late maturity status. As such, the *y*-axis was inverted for consistency with SA-CA differences.

## Results

Sex-specific descriptive statistics for age, body size, SA, and predicted estimates of maturity status and timing are summarized in Table 1. SA approximates CA, on average, in males, but is slightly in advance of CA in females. The trends in the SA-CA relationship are also suggested in the *z*-scores for the percentage of predicted adult height in boys but not in girls.

**Table 1:** Descriptive statistics for growth and maturity characteristics of male and female elite youth British tennis players

	Male (*n* = 40) (mean ± SD)	Female (*n* = 31) (mean ± SD)
Chronological age (CA), years	12.5 ± 1.8	12.7 ± 2.0
Weight, kg	48.0 ± 12.9	48.2 ± 10.6
Height, cm	159.0 ± 14.4	156.7 ± 9.7
Sitting height, cm	82.5 ± 7.4	83.0 ± 5.2
Estimated leg length, cm	76.5 ± 7.5	73.7 ± 5.1
Skeletal age (SA), years	12.7 ± 2.5	13.1 ± 2.1
SA minus CA, years	0.13 ± 1.11	0.35 ± 1.02
Father’s height, cm	182.5 ± 5.8	178.7 ± 5.7
Mother’s height, cm	168.1 ± 5.5	165.6 ± 4.9
Predicted adult height, cm	182.8 ± 5.5	167.1 ± 3.9
Height as % of adult height, %	86.9 ± 6.9	93.62 ± 5.8
Height as % of adult height, *z*	0.22 ± 0.6	− 0.11 ± 0.6
Predicted maturity offset, years	− 0.9 ± 1.8	0.8 ± 1.6
Predicted age at peak height velocity, years	13.4 ± 0.5	12.0 ± 0.6

The cross-tabulation of maturity status classifications of the two predicted indicators relative to SA indicators is summarized in Table 2. Based on SA, the majority of players of both sexes are classified as average or on time in maturity status; nine boys (23%) and eight girls (26%) are classified as early, while four (10%) boys and two (6%) girls are classified as late. In contrast, the overwhelming majority of players of both sexes are classified as on time with each predictor of maturity status, percentage of predicted adult height (88%) and predicted age at PHV (90%). No players are classified as early maturing with predicted age at PHV; few players are classified as late maturing with the two predictions, while four boys and one girl are classified as early maturing with the percentage of predicted adult height.

**Table 2:** Cross-tabulations of players by maturity status classifications based on SA and the two predicted estimates^1^

		Males				Females			
		Skeletal age				Skeletal age			
		Late	On time	Early	Total	Late	On time	Early	Total
Percentage of predicted adult height	Late	0	1	0	1	0	2	0	2
	On time	4	25	6	35	2	19	7	28
	Early	0	1	3	4	0	0	1	1
	Total	4	27	9	40	2	21	8	31
Predicted age at PHV	Late	0	0	2	2	0	2	0	2
	On time	4	27	7	38	2	19	8	29
	Early	0	0	0	0	0	0	0	0
	Total	4	27	9	40	2	21	8	31

^1^Criteria for defining maturity status with each indicator are indicated in the text

Concordance of maturity classifications for specific pairs of indicators is moderate, 61 to 70%, but Kappa coefficients are low between classifications based on SA and percentage of predicted adult height, 0.22 (*p* < 0.05) in boys and 0.05 in girls, and between classifications based on SA and predicted age at PHV, 0.08 in boys and − 0.11 in girls. Spearman’s rank order correlations between classifications based on SA and the two predicted indicators are moderate and significant in boys, 0.35 (*p* < 0.05) for SA and percentage of predicted adult height and − 0.37 (*p* < 0.05) for SA and predicted age at PHV. Corresponding Spearman’s correlations for girls are lower and not significant, 0.25 and 0.11, respectively.

**Fig. 1:**
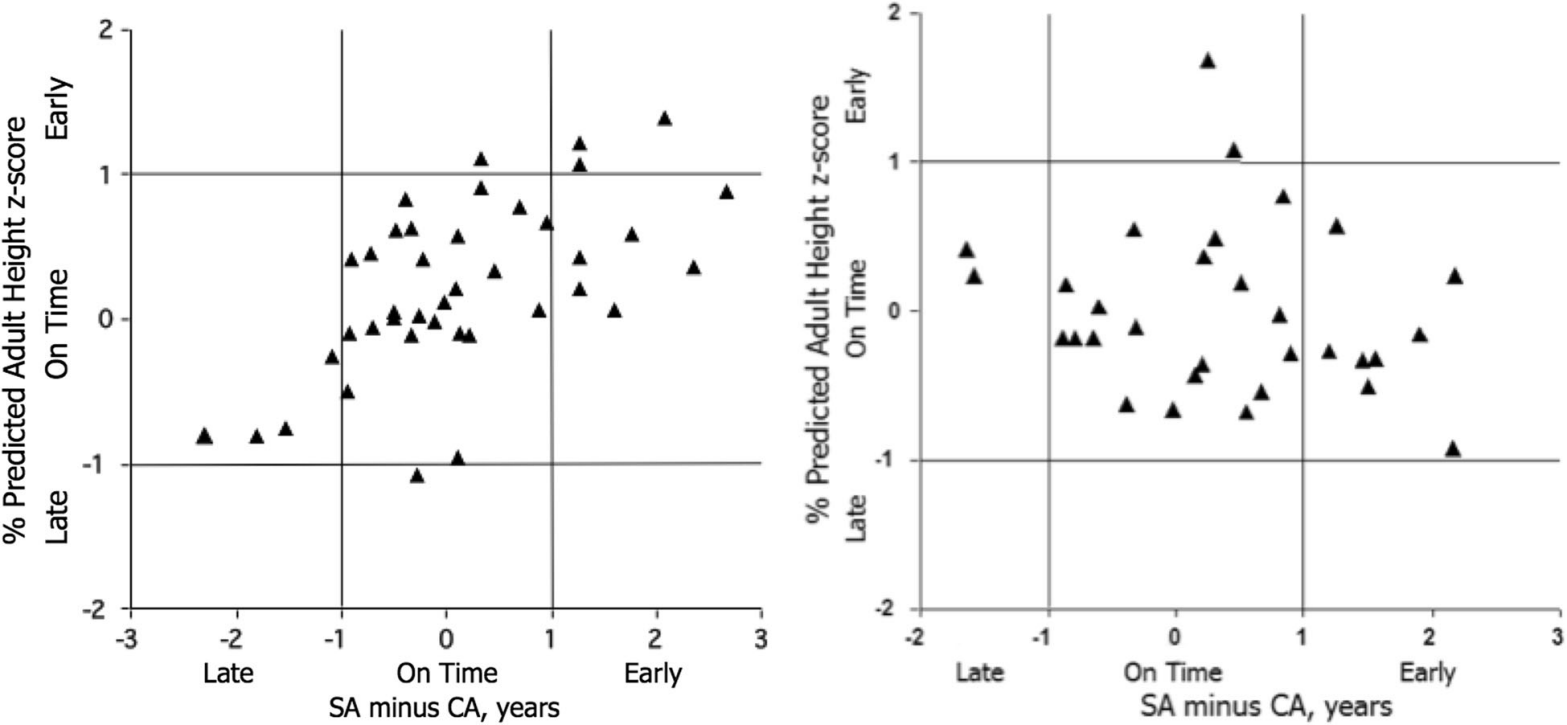
Scatter plots of absolute SA minus CA differences and *z*-scores for the percentage of predicted adult height at the time of observations in boys (left) and girls (right)

**Fig. 2:**
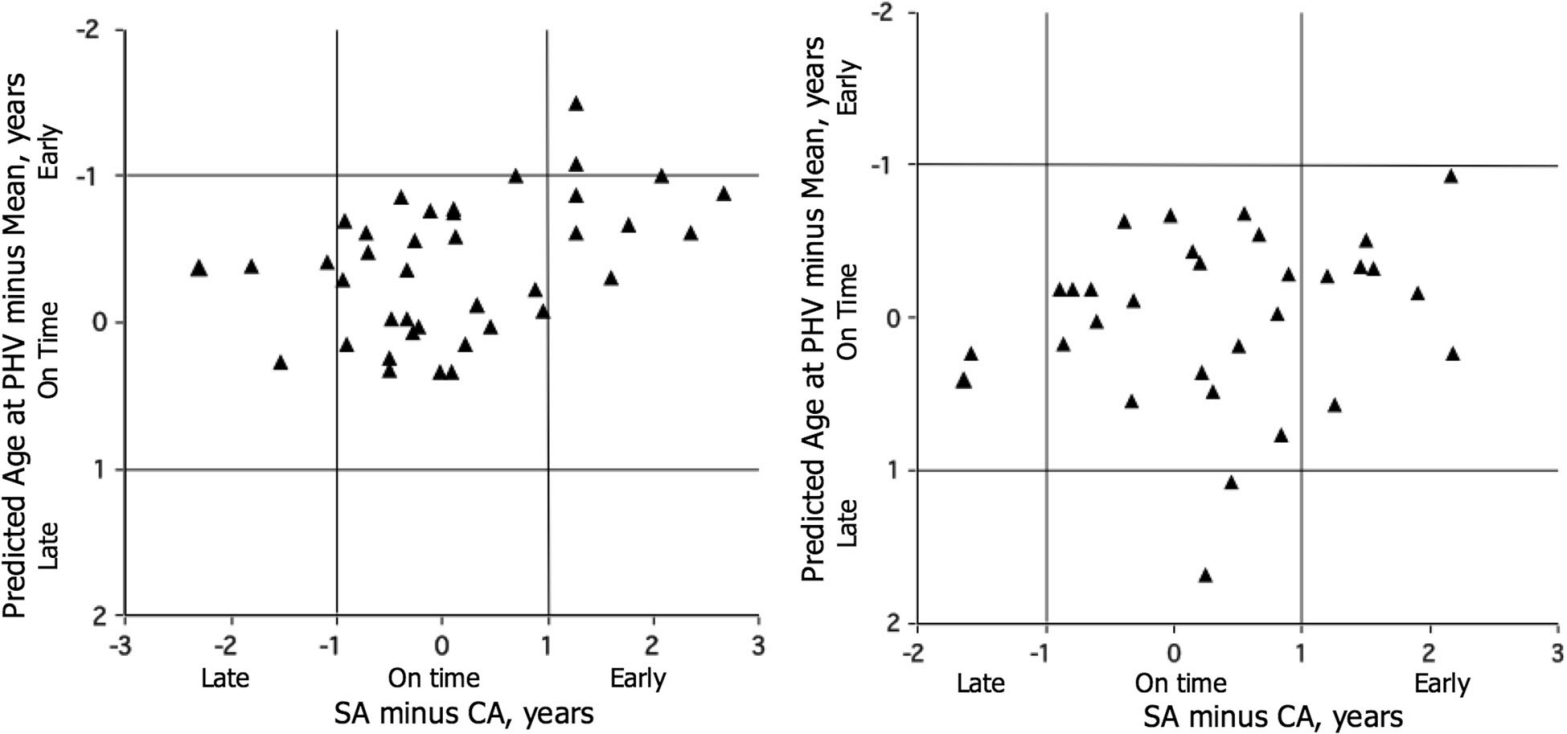
Scatter plots of absolute differences of SA minus CA and the differences of predicted age at PHV minus the sex-specific reference mean ages at PHV in boys (left) and girls (right). Note the *y*-axis is inverted, as a negative score for the difference of the predicted age at PHV minus the respective reference mean indicated early maturity status, and a positive score indicated late maturity status

Scatter plots of absolute SA-CA differences and *z*-scores for the percentage of predicted adult height are shown in Fig. 1, and of SA-CA differences and differences between predicted age at PHV and the sex-specific reference means are shown in Fig. 2. The cutoff points for defining maturity status with each indicator are also noted in the graphs. Spearman’s rank order correlations for absolute values for both indicators are moderate and significant in boys, 0.58 (*p* < 0.001) for SA and percentage of predicted adult height and 0.40 (*p* < 0.01) for SA and predicted age at PHV. Corresponding correlations for both absolute indicators are lower, and one is of marginal significance in girls, respectively, 0.32 (*p* = 0.08) and 0.18.

Corresponding analyses for players in more narrowly defined CA groups may be of interest to those working in youth tennis, where players are generally categorized into age groups. Results are summarized in Additional file 1: Tables S1, Additional file 2: Tables S2, Additional file 3: Tables S3, and Additional file 4: Table S4. Players were grouped as U10 and U12 (24 males, 17 females) and U14 and U16 (16 males, 14 females). Allowing for smaller samples in each group, percentage concordance between maturity status classifications based on SA and the two predicted indicators range from 50 to 71% for predicted age at PHV and from 63 to 81% for the percentage of predicted height. Kappa coefficients are consistently low, − 0.17 to 0.21, with the exception of a moderate coefficient (0.56, *p* < 0.05), between SA and percentage of predicted adult height among 16 male tennis players U14 and U16 years (Additional file 2: Table S2). The Spearman’s rank order correlation is also moderate and significant for this age group (0.62, *p* < 0.05). Among U10 and U12 male players, the Spearman rank order correlation is moderate and negative between classifications based on SA and predicted age at PHV (− 0.40, *p* < 0.06), while the other correlation in the younger age group approximates zero (Additional file 1: Table S1).

Spearman’s rank order correlations could not be calculated for females, as all players were classified as average based on predicted age at PHV in U10 and U12 players (*n* = 17) and percentage of predicted adult height in U14 and U16 players (*n* = 14). While the correlation is moderate (0.40, ns) for classifications based on SA and percentage of predicted adult height in U10 and U12 players (Additional file 3: Table S3), it approaches zero (0.06, ns) for classifications based on SA and predicted age at PHV in U14 and U16 female players (Additional file 4: Table S4).

## Discussion

The present study considered the appropriateness of maturity status classifications based on currently used predictive methods among elite male and female tennis players of Caucasian ancestry. Overall, the concordance of maturity status classifications based on a clinically established maturity indicator, SA, and two currently popular non-invasive predictions of maturity status was generally poor. The results suggested that the non-invasive indicators were limited in their ability to classify elite youth tennis players by maturity status, specifically at the extremes (late, early) of the maturity status continuum. Nevertheless, the results of this research suggest that the maturity status based on the percentage of predicted mature height at the time of observation correlated better with maturity status based on SA than did maturity status based on predicted age at PHV.

The moderate concordance of classification maturity status based on SA and the two predicted indicators of maturity status among the tennis players was due, in a large part, to the proportionally high numbers of players classified as average or on time (Table 2). The low kappa coefficients and at best moderate Spearman’s rank order correlations (rho) were likely a result of the small number of players classified as early or on time with each method, respectively. As in other studies comparing the maturity status of youth athletes [5, 20], a window of plus/minus 1 year was used to define average or on-time status. The window allows for errors associated with the assessment of SA and with measurements of body dimensions used in the prediction equations, in addition to errors associated with the prediction equations per se (below). Some studies, however, have used narrower bands for SA, for example, a band of ± three months among adolescent boys and girls [30] and a band of ± 0.5 year in 14-year-old soccer players [31]. The former used the TW2 method, while the latter used the TW3 method of SA assessment. Of relevance, SAs with the TW3 method were consistently less than SAs with the TW2 method by about one year in a large international sample of youth soccer players 11–17 years of age [23]. The systematic difference between TW2 and TW3 SAs has major implications for the classification of players by maturity status.

Standard errors of the estimate of SA assessments with the Fels method in the present study ranged from 0.27 to 0.60 year and were similar to the range of standard errors, 0.27 to 0.70 year, noted in three series of soccer players 11–17 years [5, 32, 33]. Higher errors were noted among youth approaching skeletal maturity. Unfortunately, both versions of the TW method and the Greulich-Pyle method do not provide an estimate of error associated with assessments of SA.

Standard errors of estimate for the maturity offset prediction equations in the current sample were 0.28 year in boys and 0.29 year in girls, which were comparably lower than the 0.59 year in boys and 0.57 year in girls obtained with prediction equations [11]. The 50% error bounds for the height prediction protocol across 4 to 17 years were, on average, 2.2 ± 0.6 cm in boys and 1.7 ± 0.6 cm in girls, while the mean 90% error bounds were, on average, 5.3 ± 1.4 cm in boys and 4.3 ± 1.6 cm in girls [14]. The error bounds, however, varied with CA. Among boys, the median 50th and 90th percentile error bounds for height predictions without SA were stable 9 to 11 years, increased from 12 to 14 years, and then declined to 16 years. The corresponding error bounds for girls increased from 9 through 12 years and then declined to 16 years. Given the preceding observations for SA and for predicted maturity offset and adult height, the band of plus/minus 1 year to define maturity groups allowed for errors associated with assessments and predictions.

The results for the relatively small sample of elite male tennis players were generally consistent with corresponding studies of participants in club-level soccer [19] and community-level American football [5], which used the same methodology (Table 3). Maturity status classifications based on predicted age at PHV had moderate concordance with classifications based on SA (Fels method) in youth soccer players, but kappa coefficients were low, 0.11 and 0.13 [19], as in the present study. Classifications of maturity status based on percentage of predicted adult height [14] also had moderate concordance with classifications based on SA (Fels method) in American football [5] and soccer [19] players. Kappa coefficients were fair to moderate (0.31 to 0.50) and varied with age in the football players in contrast to observations in tennis players and soccer players, among whom Kappa coefficients were 0.23 in both 11 to 12- and 13 to 14-year-olds. Corresponding data for female athletes in different sports are lacking.

**Table 3:** Distributions of male athletes in three sports by maturity status defined by the same indicators as in the present study

		Maturity status		
		Late	On time	Early
Skeletal age				
Tennis (9.88–15.95 years, *n* = 40)	*n*	4	27	9
	%	10	67	23
American football (9.27–14.24 years, *n* = 143)	*n*	15	52	76
	%	11	36	53
Soccer (10.98–15.25 years, *n* = 180)	*n*	21	100	59
	%	12	56	33
Percentage of predicted adult height				
Tennis	*n*	1	35	4
	%	2	88	10
American football	*n*	6	91	46
	%	4	64	32
Soccer	*n*	5	131	44
	%	3	73	24
Predicted age at PHV				
Tennis	*n*	2	38	0
	%	5	95	0
Soccer	*n*	15	161	4
	%	8	89	2

Criteria for defining maturity status with each indicator are indicated in the text and were the same in each study. Frequencies and percentages for the total samples American football and soccer players were calculated after Malina et al. [5, 19]

Predicted ages at PHV in the male and female tennis players were, respectively, 13.4 ± 0.5 years and 12.0 ± 0.6 years (Table 1). Except for an estimated age at PHV of 11.6 ± 0.9 years in 16 Japanese female tennis players who were successful at the prefecture level [34], data for ages at PHV based on longitudinal data for tennis players are lacking. The mean predicted ages at PHV for the tennis players were within the range of observed ages at PHV in several longitudinal samples of European athletes in several sports, except for gymnasts of both sexes [4]. However, standard deviations for predicted ages at PHV among tennis players were lower, 0.5 in males and 0.6 in females, but consistent with reduced standard deviations also reduced ranges of variation in predicted ages at PHV in validation studies of the maturity offset prediction protocol [10, 35, 36], and also in a longitudinal sample of Belgian artistic gymnasts [37].

Other limitations of the maturity offset prediction equations should be noted. Based on two validation studies of the original [11] and modified [38] equations, predicted maturity offset decreased while age at PHV increased, on average, with CA at prediction [10, 13, 35, 36]. This likely reflected CA per se and CA-associated variation in the predictors—sitting height, leg length, height, and weight. Of potential interest, tennis apparently selects for height; the current sample of elite players of both sexes was, on average, taller than reference values [7]. In addition, predicted ages at PHV also showed considerable intraindividual variation with CA in the validation studies and had major limitations among late and early maturing youth defined by observed age at PHV. Predicted ages at PHV were consistently later than observed in early maturing and generally earlier than observed in late maturing youth of both sexes [10, 13, 35, 36].

It also should be noted that the protocol for predicting adult height [14] and maturity offset prediction equations [11] have not been validated with UK samples. Nevertheless, allowing for the complexity of biological maturation during adolescence and inter- and intraindividual variation in the maturity status and timing of different systems, it is likely that no one system provides a complete picture of maturity status at the time of observation [39]. It would, therefore, seem prudent to compare and contrast results of the methods used in the present study with additional measures, for example, estimated growth velocities for height assuming longitudinal measures are available, and stage of pubertal development (menarche in girls, secondary sexual characteristics in boys). These would seemingly improve the accuracy of prediction methods. It would be ideal to have longitudinal growth data for youth athletes that span adolescence. Nevertheless, the inability to account for the timing or intensity of the adolescent growth spurt in height is a major source of error in height prediction equations [40], and as noted above, predicted ages at PHV with the maturity offset protocol vary significantly with CA and height at prediction [10, 13, 35, 36].

The estimates of maturity status employed in the current study assess different aspects of the maturation process. Skeletal age assesses the maturity status of the skeletal system at the time of observation, while the percentage of predicted adult height at the time of observation and predicted age at PHV are indices of, respectively, somatic maturity status and timing and are dependent upon CA, body size-attained, and proportions [19]. It is likely that the maturation of different biological systems, specifically the neuroendocrine system and the differential timing of growth spurts in height, weight, and leg and trunk length, may influence the correspondence among the three methods [19]. The maturation of the different biological systems, though related, does not proceed simultaneously.

Limitations of the current study should be noted. The elite nature of the sample, practical constraints (number and availability), and prohibitive assessment costs (skeletal hand wrist x-rays) provided challenges in obtaining ample sample sizes [41]. The results are thus specific to a relatively small sample of British elite junior tennis players of Caucasian ancestry. Generalization to players from other countries or of other ethnicities and to players competing at different levels needs to be done with care. The small sample sizes have likely limited the statistical power of the analyses conducted. Classifying players as early, on time, or late with different methods (years, *z*-scores, standard deviations) and the broad CA range of the sample (9 to 16 years) may influence the sensitivity of the maturity indicators and in turn affect the number of players assigned to each category. The band of ± one year of SA from CA within single-year CA groups is commonly used, although the range of observed standard deviations in studies of SA varies somewhat with CA and also among samples and with the method of assessment [23]. Nevertheless, maturity status classifications of players within two year competitive age groups (Additional file 1: Table S1, Additional file 2: Table S2, Additional file 3: Table S3, Additional file 4: Table S4) suggested similar trends, but numbers were limited. The limitations of using reported in contrast to measured heights of biological parents should also be noted, although research performed on a British population has indicated that average bias is less than 1 cm [42].

## Conclusions

The aim of this study was to investigate the concordance of two predictors of biological maturity status with an established method (SA) in elite youth tennis players. While the three indicators considered were interrelated, there was a relatively poor agreement between maturity classifications based on SA and those based on the two somatic indicators. Although the somatic indicators provide an inexpensive, practical, and less invasive means of measurement, the limitations of the protocols should be recognized. Predicted age at PHV with the maturity offset protocol is an especially problematic indicator, which would suggest caution when applying it to samples of youth athletes. An organization may consider using several methods of prediction in order to enhance the assessment of growth and maturity status and perhaps maturity timing. In conclusion, future research in this area should examine non-invasive methods relative to SA with longitudinal data in order to better establish the reliability and validity of the predictive methods.

## Additional Files

Additional file 1: **Table S1.** Cross-tabulations of maturity status classifications^1^ between specific pairs of maturity indicators, percentage concordance, Cohen’s kappa, and Spearman’s rank order correlations in U10 and U12 male tennis players. (PNG 154 kb)

Additional file 2: **Table S2.** Cross-tabulations of maturity status classifications^1^ between specific pairs of maturity indicators, percentage concordance, Cohen’s kappa, and Spearman’s rank order correlations in U14 and U16 male tennis players. (PNG 156 kb)

Additional file 3: **Table S3.** Cross-tabulations of maturity status classifications^1^ between specific pairs of maturity indicators, percentage concordance, Cohen’s kappa, and Spearman’s rank order correlations in U10 and U12 female tennis players. (PNG 161 kb)

Additional file 4: **Table S4.** Cross-tabulations of maturity status classifications^1^ between specific pairs of maturity indicators, percentage concordance, Cohen’s kappa, and Spearman’s rank order correlations in U14 and U16 female tennis players. (PNG 162 kb)

## Data Availability

The data that support the findings of this study are available from the Lawn Tennis Association, but restrictions may apply to their availability, which were used under license for the current study, and so are not publicly available. Data are, however, available from the authors upon reasonable request and with permission of the Lawn Tennis Association.

## Abbreviations

CA: Chronological age
PHV: Peak height velocity
rho: Spearman’s rank order correlations
RUS: Radius-ulna-short bone
SA: Skeletal age
SD: Standard deviation
TW2: Tanner-Whitehouse II
TW3: Tanner-Whitehouse III
