# Supplementary figures and images for: Cross-Sectional Analysis Investigating the Concordance of Maturity Status Classifications in Elite Caucasian Youth Tennis Players
Source: Sports Med Open. 2019 Jul 1;5:27. doi: 10.1186/s40798-019-0198-8 (PMC6603099; doi:10.1186/s40798-019-0198-8)

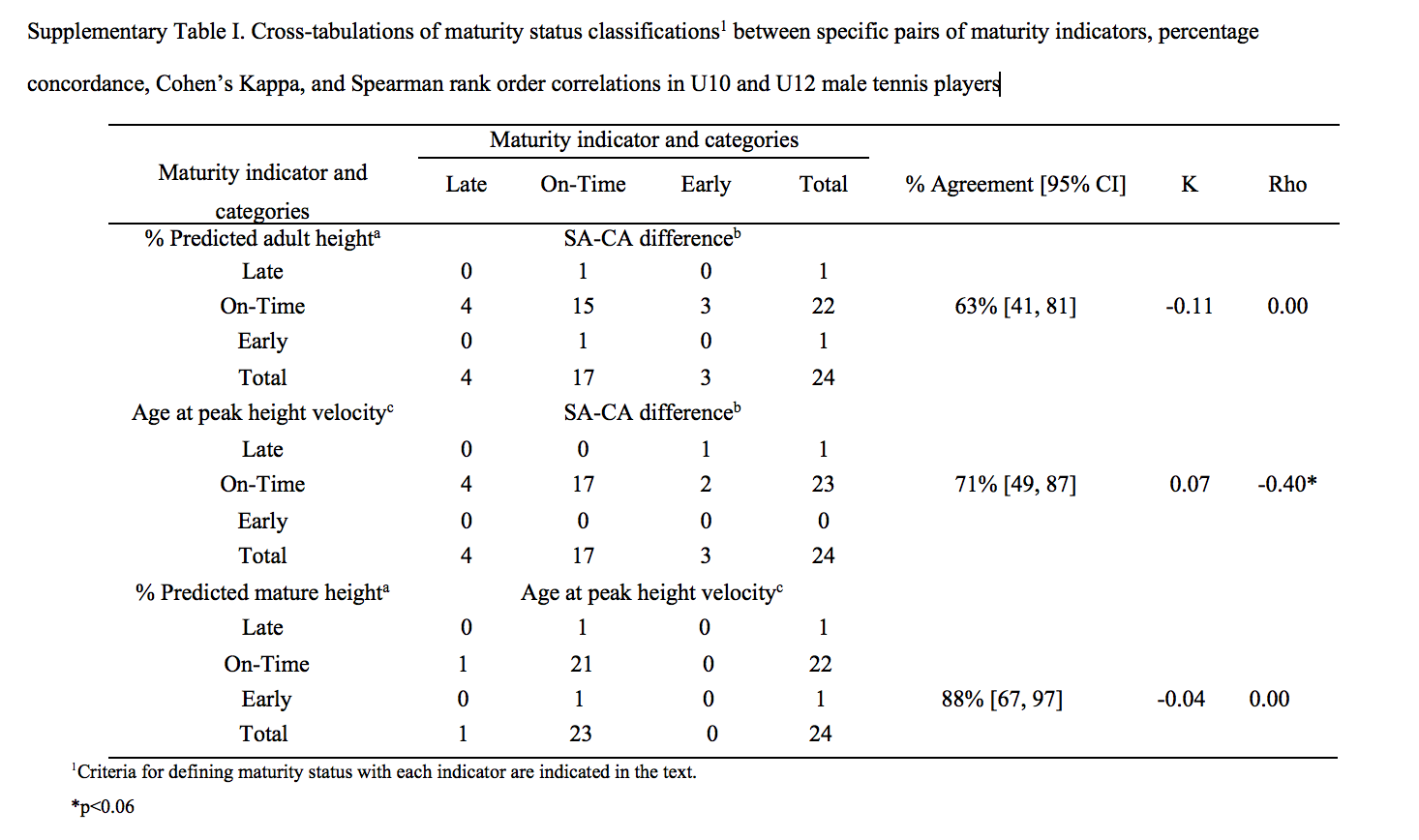

Supplement: Supplementary file 1 — Table S1. Cross-tabulations of maturity status classifications1 between specific pairs of maturity indicators, percentage concordance, Cohen’s kappa, and Spearman’s rank order correlations in U10 and U12 male tennis players. (PNG 154 kb) [file 40798_2019_198_MOESM1_ESM.png]

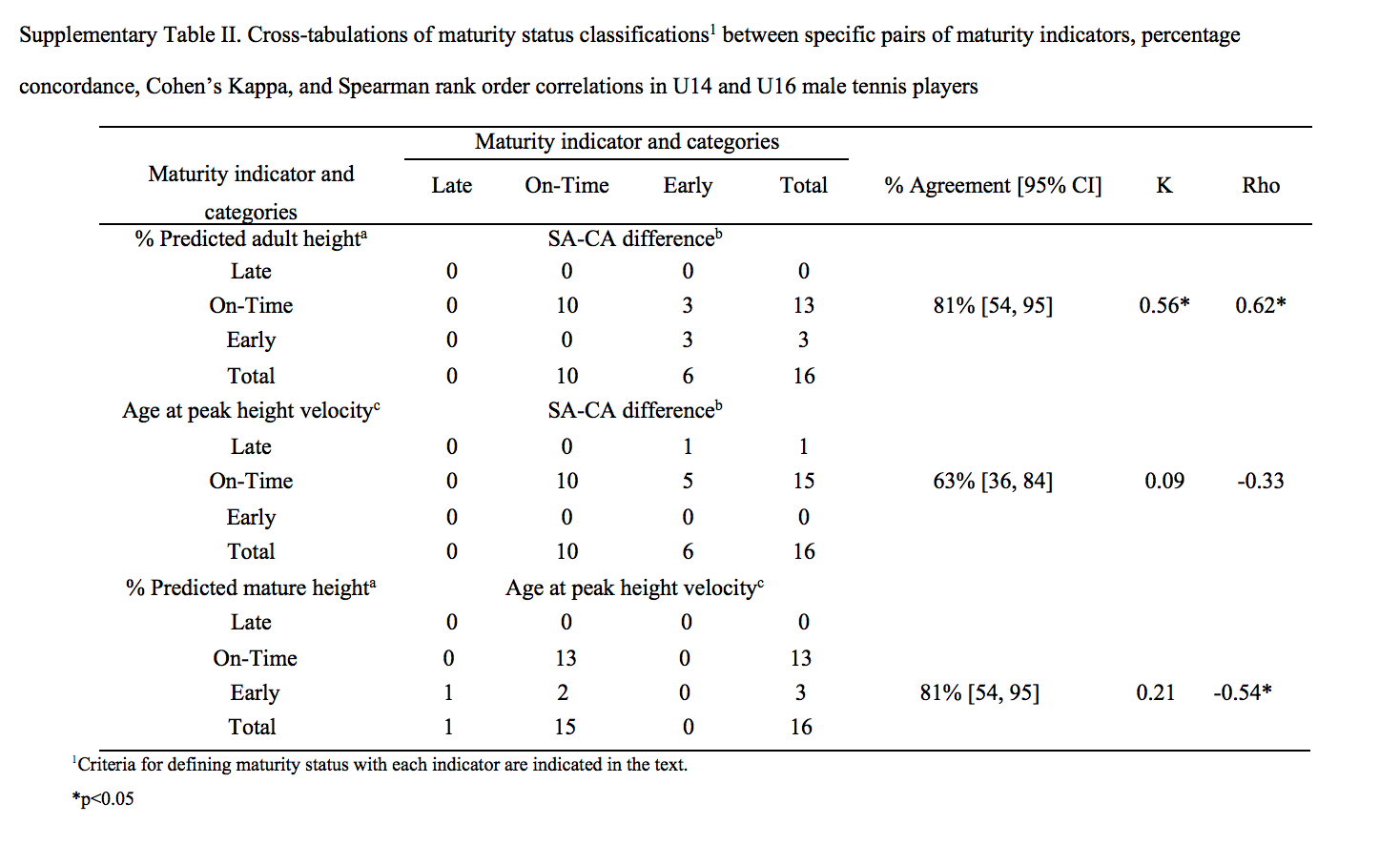

Supplement: Supplementary file 2 — Table S2. Cross-tabulations of maturity status classifications1 between specific pairs of maturity indicators, percentage concordance, Cohen’s kappa, and Spearman’s rank order correlations in U14 and U16 male tennis players. (PNG 156 kb) [file 40798_2019_198_MOESM2_ESM.png]

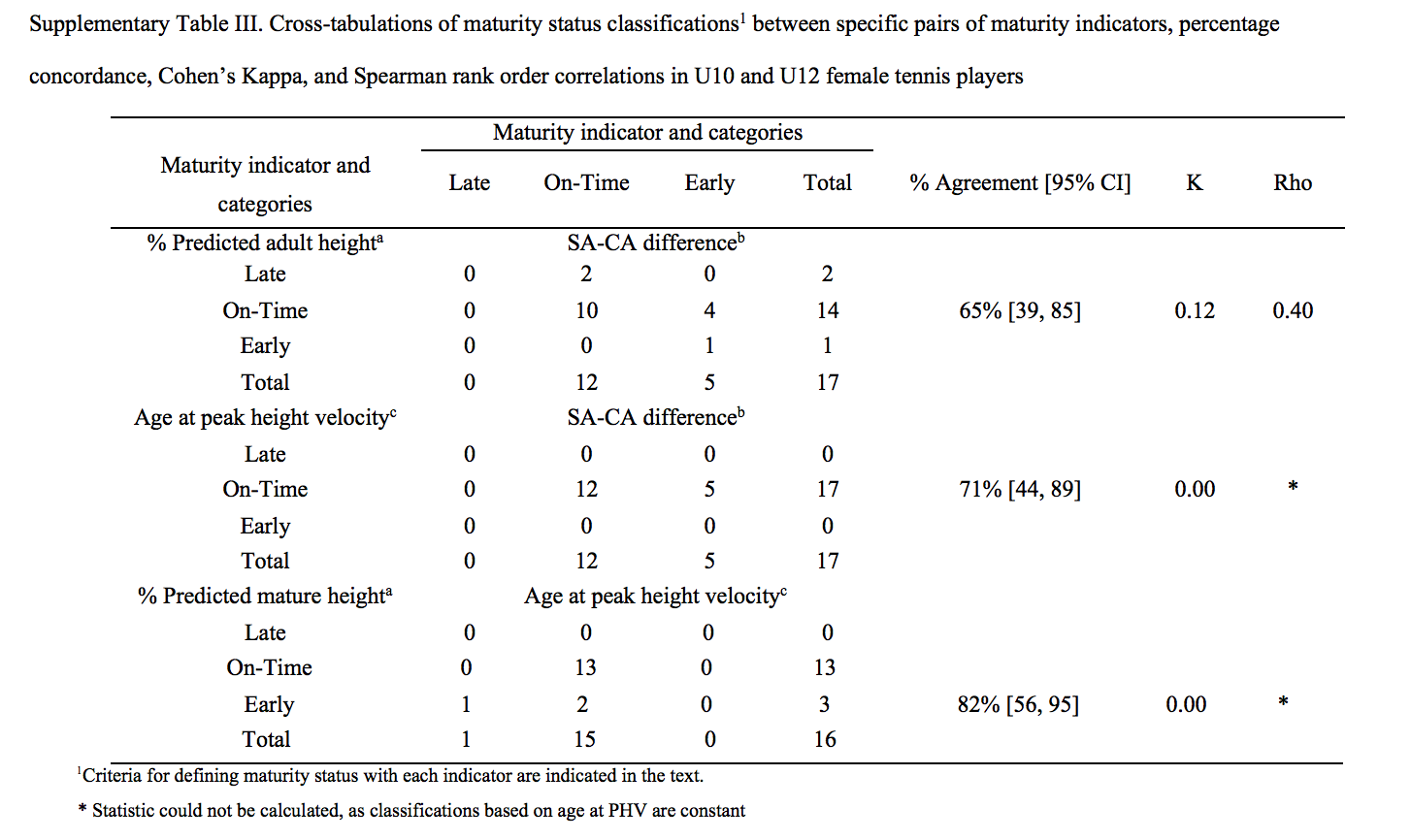

Supplement: Supplementary file 3 — Table S3. Cross-tabulations of maturity status classifications1 between specific pairs of maturity indicators, percentage concordance, Cohen’s kappa, and Spearman’s rank order correlations in U10 and U12 female tennis players. (PNG 161 kb) [file 40798_2019_198_MOESM3_ESM.png]

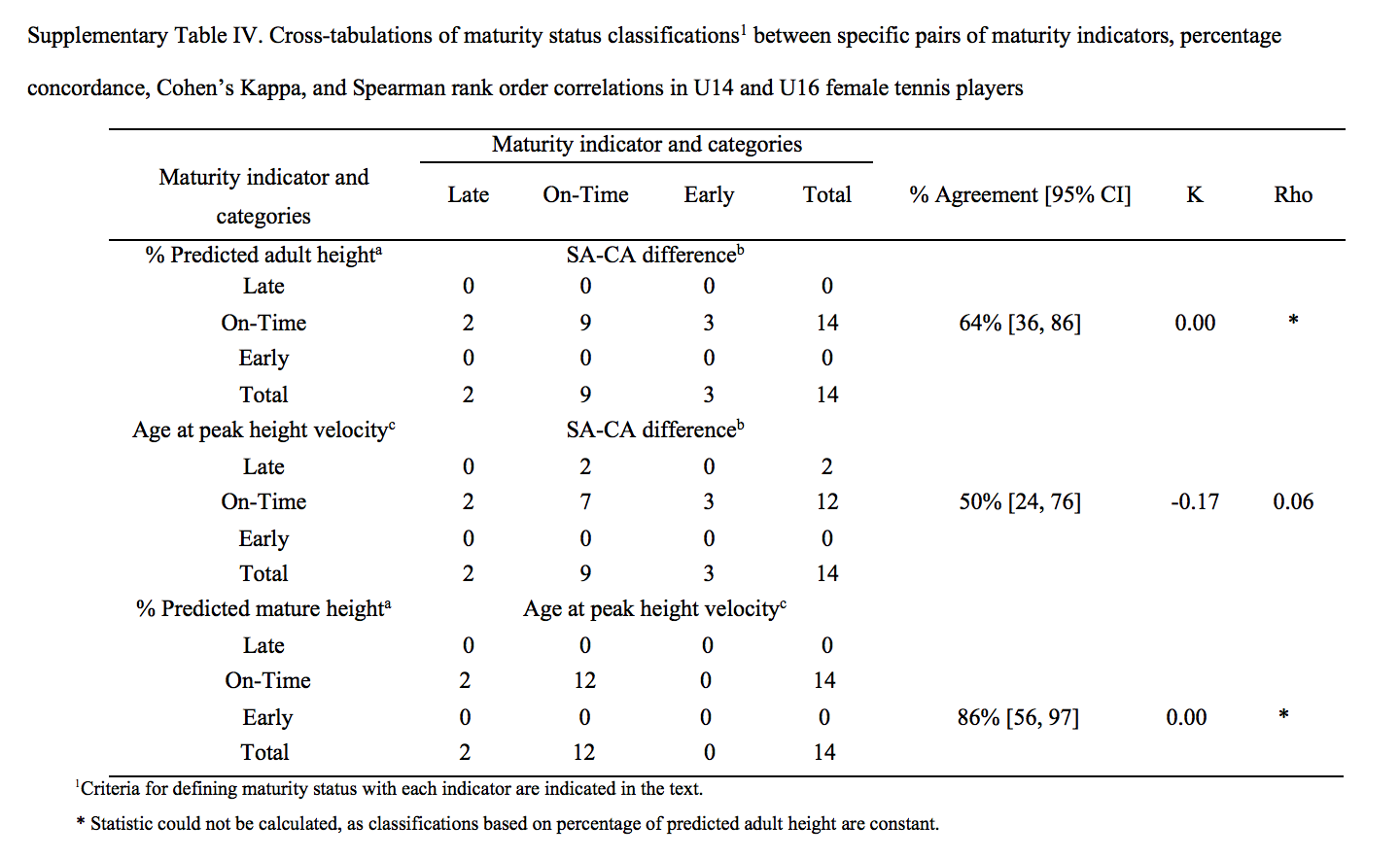

Supplement: Supplementary file 4 — Table S4. Cross-tabulations of maturity status classifications1 between specific pairs of maturity indicators, percentage concordance, Cohen’s kappa, and Spearman’s rank order correlations in U14 and U16 female tennis players. (PNG 162 kb) [file 40798_2019_198_MOESM4_ESM.png]
